# Supplementary material for: From sequence to enzyme mechanism using multi-label machine learning
Source: BMC Bioinformatics. 2014 May 19;15:150. doi: 10.1186/1471-2105-15-150 (PMC4229970; doi:10.1186/1471-2105-15-150)
Supplement: Additional file 2 — Java code of ml2db. Additional file ml2db_code.tar.gz contains the Java source code to run the multi-label machine learning experiments and save the results to database. The code’s Javadoc is included. [file 1471-2105-15-150-S2.zip › additional file 2/ml2db/ml2db/doc/main/ml2db/weka/DataSetGenerator.html]

DataSetGenerator


JavaScript is disabled on your browser.


- Overview
- Package
- Class
- Use
- Tree
- Deprecated
- Index
- Help

- Prev Class
- Next Class

- Frames
- No Frames

- All Classes

- Summary:
- Nested |
- Field |
- Constr |
- Method

- Detail:
- Field |
- Constr |
- Method


main.ml2db.weka

## Class DataSetGenerator

- java.lang.Object
- - main.ml2db.weka.DataSetGenerator

- Direct Known Subclasses:
  :   MulanDataSetGenerator

  ---

    

  ```
  public class DataSetGenerator
  extends java.lang.Object
  ```

  Generates and fills the Weka instances. The dataset can be (1) generated
  (constructor), (2) generated and filled (stores Weka instances), or (3)
  generated, filled and written to file.

  Version:
  :   3 Sep 2010

  Author:
  :   Luna De Ferrari ldf(at)st-andrews.ac.uk

- - ### Constructor Summary

    Constructors

    Constructor and Description

    `DataSetGenerator(DataSetManager manager,
    InstAttValueMap instanceAttributeMap,
    InstAttValueMap instanceClassMap)`
  - ### Method Summary

    Methods

    | Modifier and Type | Method and Description |
    | --- | --- |
    | `void` | `fillInstances()` If size is judged massive for the current hardware, while filling, write instances one by one to temp file. |
    | `java.util.Vector<weka.core.Attribute>` | `getAttributes()` |
    | `AttributeFactory` | `getAttributesFactory()` |
    | `java.lang.String` | `getClassName()` |
    | `InstAttValueMap` | `getInstanceAttributeMap()` |
    | `InstAttValueMap` | `getInstanceClassMap()` |
    | `weka.core.Instances` | `getInstances()` |
    | `InstancesFiller` | `getInstancesFactory()` |
    | `java.util.Vector<java.lang.String>` | `getInstancesNames()` |
    | `DataSetManager` | `getManager()` |
    | `int` | `getNumberOfAttributes()` |
    | `int` | `getNumberOfClassValues()` |
    | `int` | `getNumberOfInstanceAttributeCouples()` |
    | `int` | `getNumberOfInstanceClassCouples()` |
    | `int` | `getNumberOfInstances()` |
    | `int` | `getNumberOfInstancesFromDataMaps()` |
    | `int` | `getNumberOfInstancesWithClass()` |
    | `java.lang.String` | `getTempFile()` |
    | `void` | `makeInstances()` an array of zeros as long as the number of attributes, to reset all nominal (binary) attribute values of an instance to 0 (false,absent) |
    | `void` | `setInstancesNames()` The instances are all instances with a class + all instances with at least one attribute value. |

    - ### Methods inherited from class java.lang.Object

      `equals, getClass, hashCode, notify, notifyAll, toString, wait, wait, wait`

- - ### Constructor Detail


    - #### DataSetGenerator

      ```
      public DataSetGenerator(DataSetManager manager,
                      InstAttValueMap instanceAttributeMap,
                      InstAttValueMap instanceClassMap)
      ```
  - ### Method Detail


    - #### fillInstances

      ```
      public void fillInstances()
      ```

      If size is judged massive for the current hardware, while filling, write
      instances one by one to temp file.


    - #### getAttributes

      ```
      public java.util.Vector<weka.core.Attribute> getAttributes()
      ```


    - #### getAttributesFactory

      ```
      public AttributeFactory getAttributesFactory()
      ```


    - #### getClassName

      ```
      public java.lang.String getClassName()
      ```


    - #### getInstanceAttributeMap

      ```
      public InstAttValueMap getInstanceAttributeMap()
      ```


    - #### getInstanceClassMap

      ```
      public InstAttValueMap getInstanceClassMap()
      ```


    - #### getInstances

      ```
      public weka.core.Instances getInstances()
      ```


    - #### getInstancesFactory

      ```
      public InstancesFiller getInstancesFactory()
      ```


    - #### getInstancesNames

      ```
      public java.util.Vector<java.lang.String> getInstancesNames()
      ```


    - #### getManager

      ```
      public DataSetManager getManager()
      ```


    - #### getNumberOfAttributes

      ```
      public int getNumberOfAttributes()
      ```


    - #### getNumberOfClassValues

      ```
      public int getNumberOfClassValues()
      ```


    - #### getNumberOfInstanceAttributeCouples

      ```
      public int getNumberOfInstanceAttributeCouples()
      ```


    - #### getNumberOfInstanceClassCouples

      ```
      public int getNumberOfInstanceClassCouples()
      ```


    - #### getNumberOfInstances

      ```
      public int getNumberOfInstances()
      ```


    - #### getNumberOfInstancesFromDataMaps

      ```
      public int getNumberOfInstancesFromDataMaps()
      ```

      Returns:


    - #### getNumberOfInstancesWithClass

      ```
      public int getNumberOfInstancesWithClass()
      ```


    - #### getTempFile

      ```
      public java.lang.String getTempFile()
      ```


    - #### makeInstances

      ```
      public void makeInstances()
      ```

      an array of zeros as long as the number of attributes, to reset all
      nominal (binary) attribute values of an instance to 0 (false,absent)


    - #### setInstancesNames

      ```
      public void setInstancesNames()
      ```

      The instances are all instances with a class + all instances with at
      least one attribute value.


- Overview
- Package
- Class
- Use
- Tree
- Deprecated
- Index
- Help

- Prev Class
- Next Class

- Frames
- No Frames

- All Classes

- Summary:
- Nested |
- Field |
- Constr |
- Method

- Detail:
- Field |
- Constr |
- Method
